# Effect of Extracelluar Vesicles Derived from *Akkermansia muciniphila* on Intestinal Barrier in Colitis Mice

**DOI:** 10.3390/nu15224722

**Published:** 2023-11-08

**Authors:** Ting Zheng, Haining Hao, Qiqi Liu, Jiankun Li, Yukun Yao, Yisuo Liu, Tai Zhang, Zhe Zhang, Huaxi Yi

**Affiliations:** 1 State Key Laboratory of Marine Food Processing & Safety Control, College of Food Science and Engineering, Ocean University of China, Qingdao 266000, China; 17861506680@163.com (T.Z.); haohaining1994@163.com (H.H.); liuqiqi9306@126.com (Q.L.); lijiankun2333@163.com (J.L.); yukun_yao@163.com (Y.Y.); liuyisuo@stu.ouc.edu.cn (Y.L.); tyzhang@stu.ouc.edu.cn (T.Z.); 2 Food Laboratory of Zhongyuan, Luohe 462300, China

**Keywords:** *Akkermansia muciniphila*, extracellular vesicles, colitis, prevention, intestinal barrier, gut microbiota

## Abstract

Inflammatory bowel disease (IBD) is a chronic and recurrent disease. It has been observed that the incidence and prevalence of IBD are increasing, which consequently raises the risk of developing colon cancer. Recently, the regulation of the intestinal barrier by probiotics has become an effective treatment for colitis. *Akkermansia muciniphila*-derived extracellular vesicles (Akk EVs) are nano-vesicles that contain multiple bioactive macromolecules with the potential to modulate the intestinal barrier. In this study, we used ultrafiltration in conjunction with high-speed centrifugation to extract Akk EVs. A lipopolysaccharide (LPS)-induced RAW264.7 cell model was established to assess the anti-inflammatory effects of Akk EVs. It was found that Akk EVs were able to be absorbed by RAW264.7 cells and significantly reduce the expression of nitric oxide (NO), TNF-α, and IL-1β (*p* < 0.05). We explored the preventative effects on colitis and the regulating effects on the intestinal barrier using a mouse colitis model caused by dextran sulfate sodium (DSS). The findings demonstrated that Akk EVs effectively prevented colitis symptoms and reduced colonic tissue injury. Additionally, Akk EVs significantly enhanced the effectiveness of the intestinal barrier by elevating the expression of MUC2 (0.53 ± 0.07), improving mucus integrity, and reducing intestinal permeability (*p* < 0.05). Moreover, Akk EVs increased the proportion of the beneficial bacteria *Firmicutes* (33.01 ± 0.09%) and downregulated the proportion of the harmful bacteria *Proteobacteria* (0.32 ± 0.27%). These findings suggest that Akk EVs possess the ability to regulate immune responses, protect intestinal barriers, and modulate the gut microbiota. The research presents a potential intervention approach for Akk EVs to prevent colitis.

## 1. Introduction

Inflammatory bowel disease (IBD), which consists primarily of Crohn’s disease (CD) and ulcerative colitis (UC), is a recurrent inflammatory disease of the gastrointestinal tract [1]. The increasing prevalence of IBD has placed a heavy burden on health [2]. Intestinal barrier damage, immune dysfunction [3], and gut microbiota imbalance [4] are the significant features of IBD. Chemical, physical, immunological, and biological barriers make up the intestinal barrier. These barriers collaborate to facilitate optimal absorption and usage of nutrition while simultaneously preventing pathogenic microbes and contaminants from entering the body [5]. The progression of IBD was linked to an imbalance of the physical and biological barriers, including a loss of epithelial cell tight junctions, a rise in pathogenic bacteria, and a decrease in microbial diversity [6]. Based on these findings, the intestinal barriers have the potential to be targeted for IBD prevention. *Akkermansia muciniphila* (Akk) is a beneficial microorganism colonized in the mucous layer of the intestinal tract [7]. Accumulating results showed that Akk correlated with intestinal barrier protection [8], immunoregulation [9], and gut microbiome modulation [10]. Bacterial extracellular vesicles (BEVs) are nano-vesicles rich in various bioactive components that play a vital role in bacterial-bacterial and bacterial-host interactions [11]. *Akkermansia muciniphila*-derived extracellular vesicles (Akk EVs) have multiple regulatory effects on human health. Previous animal studies have shown that Akk EVs could ameliorate obesity by regulating intestinal homeostasis [12] and improve type 2 diabetes by promoting tight junctions [13]. Wang et al. [14] discovered that Akk outer membrane vesicles could enter intestinal epithelial cells to promote tight junctions and mucus expression to sustain the integrity of the intestinal wall. Additionally, Akk EVs alleviated nonalcoholic fatty liver disease [15] and ulcerative colitis [16] by inhibiting inflammation. Moreover, Akk outer membrane vesicles may specifically encourage the growth of advantageous bacteria via membrane fusion, thereby reestablishing the unbalanced gut microbiota [14]. Akk EVs have demonstrated the ability to produce anti-inflammatory properties, preserve the integrity of the intestinal and regulate the intestinal microbiota. However, the current studies on Akk EVs primarily focus on single aspects or the treatment of colitis symptoms. There remains a gap in research regarding the preventive effects of Akk EVs on colitis. Consequently, the focus of this investigation is to study the potential of Akk EVs in preventing colitis. In this study, whether supplementation with Akk EVs could prevent colitis by inhibiting inflammatory reactions, restoring the intestinal mucus layer, protecting the intestinal barrier, and balancing the intestinal microflora structure was explored. This study offers a potential strategy for utilizing Akk EVs as an intervention to prevent colitis.

## 2. Materials and Methods

### 2.1. Akk Culture and Akk EVs Preparation

*Akkermansia muciniphila* ATCC BAA-835 was provided by China Agricultural University (Beijing, China) and cultured in BHI (Hopebio, Qingdao, China) supplemented with 0.05% L-Cysteine hydrochloride anhydrous (Macklin, Shanghai, China) under anaerobic conditions [17]. Akk EVs isolation was carried out in the manner previously mentioned [18]. After being centrifuged at 10,000× *g* for 20 min at 4 °C, the bacterial liquid was pelleted, and then the supernatant underwent filtration using a 0.22 µm PES Vacuum Filter. The filtrate was concentrated using Vivaflow™ 50 crossflow cassettes (Sartorius, Gottingen, Germany) and subsequently ultracentrifuged at 200,000× *g* for 3 h in an MLA-50 rotor (Beckman Coulter, Brea, CA, USA) at 4 °C. After being resuspended in PBS, the particles were ultracentrifuged at 200,000× *g* for 1.5 h at 4 °C to wash them. The resultant final particles were suspended in PBS (Solarbio, Beijing, China) and frozen at −80 °C. Nanoparticle Tracking Analysis (NTA) measured the dimension range of Akk EVs, and the Transmission Electron Microscope (TEM) visualized the morphology of Akk EVs.

### 2.2. Cell Culture

The RAW264.7 cells were provided by the China Agricultural University (Beijing, China) and cultured in DMEM (Solarbio, Beijing, China) medium supplemented with 10% fetal bovine serum (Biological Industries, Kibbutz Beit Haemek, Israel) and 1% 100× Penicillin-Streptomycin Liquid (Solarbio, Beijing, China) at 37 °C in a 5% CO_2_ atmosphere [19].

### 2.3. Measurement of Cellular Uptake of Akk EVs

Using a previously outlined technique, the resuspended Akk EVs were tagged with Dil (ThermoFisher Scientific, Waltham, MA, USA) to visualize the cellular absorption of Akk EVs in vitro [20]. In summary, 1.5 mL of resuspended Akk EVs were incubated with 1.5 mL of a 5 µM Dil solution for 1 h in the dark. Any remaining free dyes were removed by ultracentrifugation at 200,000× *g* for 1.5 h. Dil-labeled Akk EVs (100 μg/mL) were subsequently cultured for 10 h with RAW264.7 cells (2 × 10^5^ cells/mL). After the incubation period, three PBS washes and the cells were fixed for 10 min using Immunol Staining Fix Solution (Beyotime, Shanghai, China). After three PBS washes, the samples were stained for 30 min in the dark using Actin Green 488 Ready Probes (ThermoFisher Scientific) DAPI was used to mark the cell nucleus (Beyotime, Shanghai, China). A laser scanning confocal microscope (Zeiss, Oberkochen, Germany) was used to observe the cellular absorption of Akk EVs.

### 2.4. Measurement of Cytokine and Nitric Oxide (NO) Production

The RAW264.7 cells (2 × 10^5^ cells/mL) were plated and induced with lipopolysaccharide (LPS, Sigma-Aldrich, St. Louis, MO, USA) dissolved in DMEM (100 ng/mL), then 20 h later cells were treated with Akk EVs (10, 50, and 100 μg/mL) [21]. In the same way, 20 h later, cells were treated with Akk (10^7^ CFU). After 24 h, the supernatant was collected and utilized for NO and cytokine testing. As per the manufacturer’s instruction, the levels of cytokines and NO were quantified using an ELISA kit and NO kit (Nanjing Jiancheng, Nanjing, China), respectively. 

### 2.5. Animals and Treatments

The specific pathogen-free (SPF) C57BL/6J male mice (6 weeks) were obtained from Charles River Laboratories (Beijing, China) and maintained under a controlled SPF animal facility (temperature of 22 ± 2 °C and 50 ± 5% of relative humidity) with a 12 h/12 h light/dark cycle. Clean water and standard food were freely given to mice. After adapting to the environment for 7 days, randomly, all mice were separated into four groups: the control group (Con), the DSS group (DSS), the Akk group (Akk), and the Akk EVs group (EVs). Specifically, from days 8–28, the control group and DSS group were given 0.2 mL PBS. The Akk group and Akk EVs group were given 0.2 mL of Akk suspension (10^9^ CFU) and 0.2 mL of Akk EVs (10 µg protein) by oral gavage once daily, respectively [22]. Three other groups were induced with 3.5% dextran sulfate sodium (DSS, 36–50 kDa molecular weight, Yeasen, Shanghai, China) between days 22 and 28, with the exception of the control group [23]. According to the previous research method, the experiment observed the mice’s state and recorded the mice’s weight, fecal traits, blood in the stool, and disease activity index (DAI). The details of DAI scoring methods are shown in Table S1 [24]. On the 29th day, all mice were dissected after anesthesia, collecting serum, cecal contents, spleen, liver, and colon tissue, and measuring colonic length for subsequent experiments. All experimental procedures conducted in this investigation were approved and monitored by the Animal Ethics Committee of Ocean University of China, with permission number SPXY 2022030801.

### 2.6. Histomorphological and Immunohistochemical Assessment

The distal colon tissues were collected and fixed for 24 h with 4% paraformaldehyde. The tissues were subsequently embedded in paraffin and sectioned at a thickness of 4 µm. Hematoxylin-Eosin (HE) and Alcian Blue Periodic Acid Schiff (AB-PAS) were used to stain the paraffin sections of each colon, respectively. To examine the presence of MUC2 and zonula occludens-1 (ZO-1), the colonic tissue paraffin sections underwent deparaffinization and rehydration. Next, antigen heat retrieval was performed by placing the samples in citric acid. Following this, the sections were treated with 3% hydrogen peroxide and incubated in the darkness for 25 min. Subsequently, 3% BSA was applied to the tissues and allowed to seal at room temperature. The primary antibody was added to the sections, which were then placed evenly in a moist box and incubated overnight for immunostaining. After shaking and drying the sections, the tissues were covered with a secondary antibody labeled with HRP (horseradish peroxidase) from the corresponding species of the primary antibody. This was followed by incubation at room temperature. Finally, 3,3′-diaminobenzidine (3,3′-DAB) was added to develop color, and hematoxylin was used to counterstain the sections. All stained samples were visualized under a microscope. The scoring standard for evaluating colon histological injury is presented in Table S2 [18].

### 2.7. Measurement of Biochemical Parameters

After collecting the mice’s blood, it was kept at room temperature for 3 h. Subsequently, the serum was separated by centrifugation at 5000× *g* for 30 min at 4 °C. ELISA kits (Nanjing Jiancheng, China) were utilized to measure the levels of TNF-α, IL-6, IL-1β, Diamine Oxidase (DAO), D-lactic acid (D-LA), Myeloperoxidase (MPO), IgA, and sIgA in the serum.

### 2.8. Quantitative Real-Time PCR

To extract total RNA from colon tissues, the FastPure Tissue Total RNA Isolation Kit V2 (Vazyme, Nanjing, China) was utilized. Reverse transcription and quantitative PCR (qPCR) were performed using the Master Mix (TOYOBO, Osaka, Japan). The StepOnePlus Real-Time PCR Instrument (ThermoFisher Scientific) was employed to quantify the expression of MUC2, ZO-1, and GAPDH genes. The 2^−ΔΔCt^ method was used to compute the genes’ relative expression. Table S3 is for the Real-Time PCR primer sequences [25,26].

### 2.9. 16S rRNA Gene Sequencing

The total DNA was obtained from four groups’ collected mouse fecal samples. The forward primer 338F (5′-ACTCCTACGGGAGGCAGCA-3′) and the reverse primer 806R (5′-GGACTACHVGGGTWTCTAAT) were used to amplify the V3–V4 regions of bacterial 16S rRNA. Sequencing was conducted using the Illumina MiSeq platform at Nanjing Personalbio (Nanjing, China). After sequencing, Genescloud software was used to analyze data.

### 2.10. Statistical Analysis

All results were recorded as means ± standard deviations (mean ± SD). Statistical analyses were performed using one-way analysis of variance (ANOVA), followed by Tukey’s test with IBM SPSS Statistics 22.0. The difference was statistically significant with *p* < 0.05. 

## 3. Results

### 3.1. Characterization of Akk EVs

**Figure 1:**
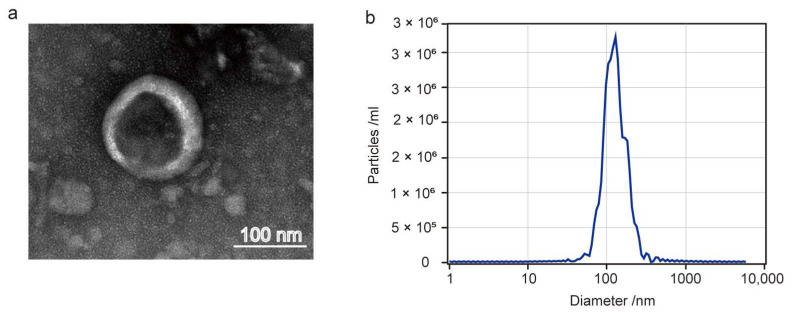
Characterization of Akk EVs. (**a**) TEM images of Akk EVs. (**b**) Size distribution of Akk EVs.

The characterizations of Akk EVs were assayed by NTA and TEM. The results indicated that Akk EVs exhibited a spherical shape and were enclosed within a double-layered membrane (Figure 1a). The size distribution of Akk EVs ranged from 30 nm to 350 nm, with a peak value of 121.6 nm observed (Figure 1b).

### 3.2. Cellular Uptake and Anti-Inflammatory Activity of Akk EVs

**Figure 2:**
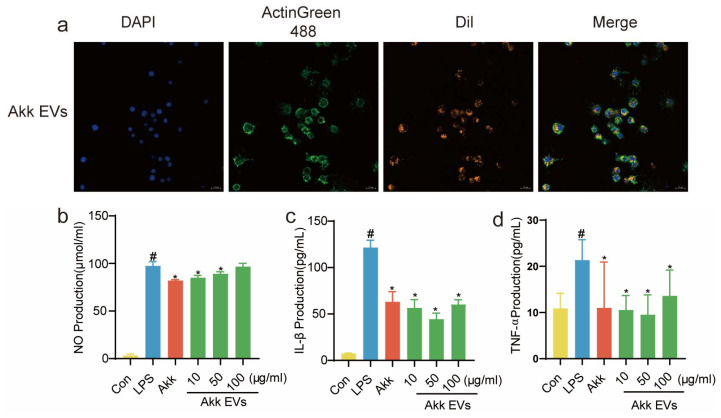
Akk EVs suppress LPS-induced inflammatory responses in RAW264.7 cells. (**a**) Fluorescence images of cell internalization profiles of Dil-loaded EVs in RAW264.7 cells. Scale bar represents 20 μm. Effects of Akk EVs on the production of NO (**b**), IL-1β (**c**), and TNF-α (**d**) in the presence of 100 ng/mL LPS in RAW264.7 cells. All data were presented as mean ± SD (*n* = 5). * *p* < 0.05, compared with the LPS group; # *p* < 0.05, compared with the control group.

The cellular absorption of Akk EVs was investigated by laser scanning confocal microscope in vitro. The Dil-labeled Akk EVs were stained orange, the nucleus was stained blue by DAPI, and the cytoskeleton was stained green by ActinGreen 488. The obvious Dil signals were observed from Akk EVs-treated RAW264.7 cells, suggesting that Akk EVs were effectively internalized by RAW264.7 cells (Figure 2a). Moreover, treatment with Akk EVs inhibited the elevated activity of NO and pro-inflammatory cytokines in LPS-activated RAW264.7 cells. In Figure 2b–d, compared with the control group, LPS significantly increased the production of NO, IL-1β, and TNF-α in RAW264.7 cells, while Akk and Akk EVs (10–50 µg/mL) expressively reduced the levels of these pro-inflammatory cytokines in contrast to the LPS group (*p <* 0.05). Furthermore, Akk EVs (10–50 µg/mL) seemed to inhibit the production of inflammatory mediators more obviously than Akk EVs (100 µg/mL). Therefore, Akk EVs could be absorbed by RAW264.7 cells and exert anti-inflammatory activity in vitro.

### 3.3. Effects of Akk EVs on the Symptoms

**Figure 3:**
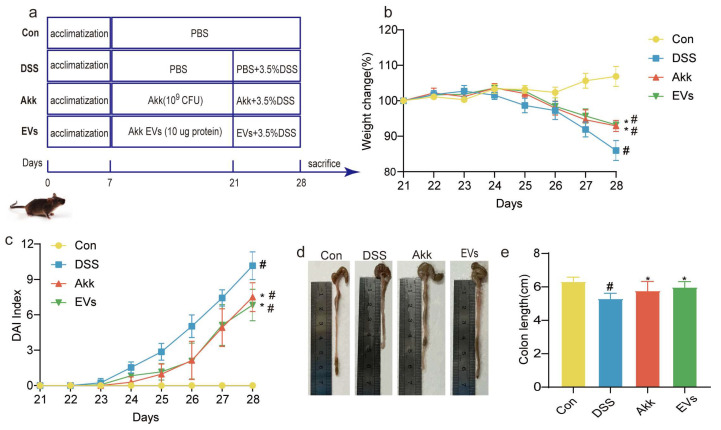
Experimental schedule and basic indicators. (**a**) Animal treatment schedule. (**b**) Bodyweight change in each group. (**c**) DAI scores in each group. (**d**) Representative images of the colon length. (**e**) Colon length. All data were presented as mean ± SD (*n* = 6). Control group (Con), DSS group (DSS), Akk group (Akk), and Akk EVs group (EVs). * *p* < 0.05, compared with the DSS group; # *p* < 0.05, compared with the control group.

To further investigate the preventive effect of oral Akk EVs on colitis in vivo, a colitis mouse model was established using 3.5% DSS (Figure 3a). As shown in Figure 3b, during the 7 days of DSS intervention, the body weight of mice in the DSS group declined when compared to that of the control group. On day 28, the body weight change of the DSS group was reduced to 85.99 ± 2.82%, while supplementation of Akk and Akk EVs significantly improved the loss of body weight, recovering to 92.91 ± 1.56% and 93.22 ± 0.38%, respectively (*p <* 0.05). In Figure 3c, during the DSS treatment, the DAI score effectively increased compared with the control group. But the Akk group (7.50 ± 1.23) and Akk EVs group (6.83 ± 1.33) markedly decreased the scores of DAI compared to the DSS group (10.17 ± 1.17) (*p <* 0.05). Additionally, in Figure 3d–e, the colon length in the DSS group (5.30 ± 0.32 cm) was significantly lower than that in the control group (6.33 ± 0.26 cm). Nevertheless, colon shortening was significantly reduced after administering Akk and Akk EVs, recovering to 5.77 ± 0.56 cm and 5.98 ± 0.33 cm, respectively (*p <* 0.05). In general, Akk and Akk EVs showed a similar effect. These findings suggest that treatment with Akk EVs may inhibit the occurrence and progression of colitis.

### 3.4. Effects of Akk EVs on Histopathological Characteristics

**Figure 4:**
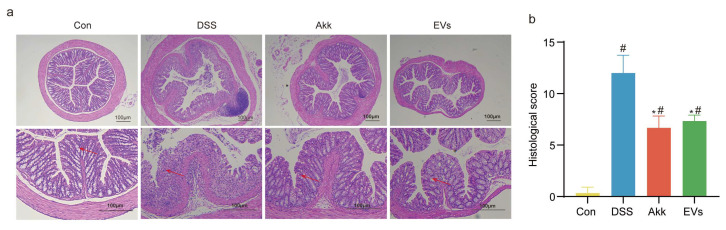
Histopathological Characteristics of colitis mice. (**a**) Typical histological sections stained with HE. (**b**) The scoring of colon histological injury. Scale bar represents 100 μm. Red arrows indicated colon lesions. Control group (Con), DSS group (DSS), Akk group (Akk), and Akk EVs group (EVs). * *p* < 0.05, compared with the DSS group; # *p* < 0.05, compared with the control group.

To evaluate colonic tissue damage, we utilized HE staining. In Figure 4a, the colon injury scores of the control group, DSS group, Akk group, and Akk EVs group were 0.33 ± 0.58, 12.00 ± 1.73, 6.67 ± 1.15, and 7.33 ± 0.58, respectively. The representative staining histological sections are shown in Figure 4b. There was no inflammatory infiltration in the colon tissue of the mice in the control group. The structure of the mucous layer, submucosa, and muscle layer appeared clear, with neatly arranged and regular-shaped recesses. Additionally, a significant number of goblet cells were clearly visible without any pathological changes. In the DSS group, there was a severe degree of inflammatory infiltration observed in the mucosa and submucosa, while the degree of inflammatory infiltration in the muscle layer was mild. The crypts displayed atrophy and deformity, with significant damage to the surface epithelium and a substantial reduction in goblet cells. The extent of the lesions accounted for more than 85% of the affected area. In contrast to the DSS group, the inflammatory infiltration in the colon tissue of mice in the Akk EVs group was only observed in the mucosal layer. The loss of crypts and goblet cells was restored, the surface epithelium remained intact, and the scope of lesions was reduced. Similarly, the Akk group also exhibited a lighter degree of inflammatory cell infiltration in the colon tissue. Overall, these findings implied that treatment with Akk EVs could relieve damage to colon tissues.

### 3.5. Effects of Akk EVs on Mucus Destruction and Goblet Cell Exhaustion

**Figure 5:**
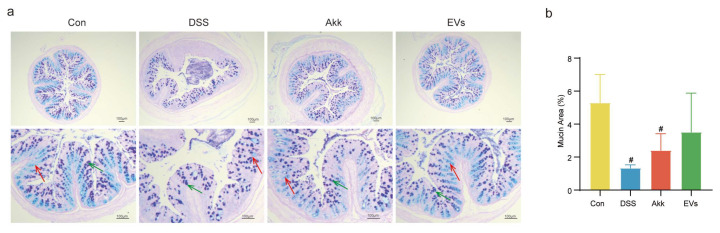
AB-PAS staining. (**a**) Typical histological sections stained with AB-PAS. (**b**) Mucin area (%). Scale bar represents 100 μm. Red arrows indicated acidic mucin and green arrows indicated mixed mucin. Control group (Con), DSS group (DSS), Akk group (Akk), and Akk EVs group (EVs). # *p* < 0.05, compared with the control group.

AB-PAS staining was used for estimating the integrity of the colonic mucus layer. In Figure 5, the distribution of the mucus layer in the control group remained intact, with a distribution area proportion of 5.30 ± 1.70%. In contrast, the DSS group exhibited a significant decrease in mucus distribution, with a proportion of mucus distribution area of 1.34 ± 0.19%. Comparatively, the mice in both the Akk group and Akk EVs group showed a recovery in the mucus area, accounting for 2.41 ± 1.00% and 3.53 ± 2.36%, respectively, when compared to the DSS group. Furthermore, the main components of the mucus were identified as acidic mucin and neutral mucin. After AB-PAS staining, acidic mucin appeared blue, neutral mucin displayed a purplish-red color, and mixed mucin exhibited a blue-purple hue. The control group displayed a substantial amount of acidic mucin, while the DSS group showed a minimal presence. However, both the Akk group and Akk EVs group demonstrated an increase in the distribution of acidic mucin in contrast to the DSS group. These results implied that Akk EVs could improve mucus destruction and increase acidic mucin.

### 3.6. Effects of Akk EVs on Immunoglobulins and Inflammatory Cytokine

**Figure 6:**
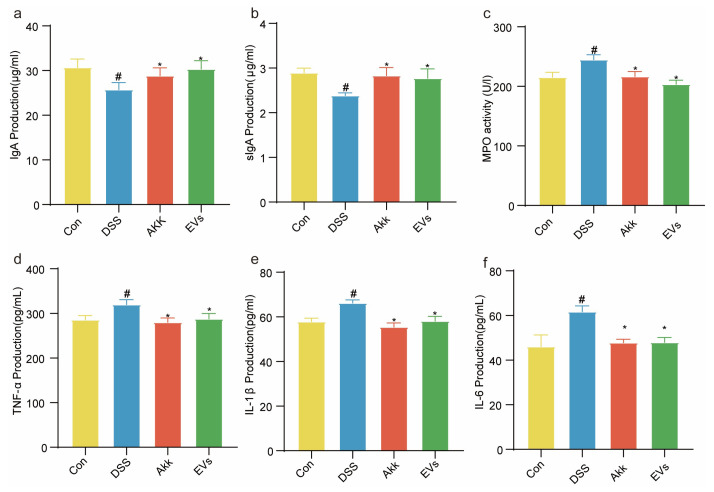
Effects of Akk EVs on immunoglobulins and inflammatory cytokine. (**a**) IgA. (**b**) sIgA. (**c**) MPO. (**d**) TNF-α. (**e**) IL-1β. (**f**) IL-6. All data were presented as mean ± SD (*n* = 6). Control group (Con), DSS group (DSS), Akk group (Akk), and Akk EVs group (EVs). * *p* < 0.05, compared with the DSS group; ^#^ *p* < 0.05, compared with the control group.

The effects of Akk EVs on IgA, sIgA, MPO, and inflammatory cytokines in mice serum were shown in Figure 6. In contrast to the control group, the concentration of IgA and sIgA in the DSS group were decreased. The concentration of MPO, TNF-α, IL-1β, and IL-6 in the DSS group was increased (*p* < 0.05). After treatment with Akk, the levels of IgA and sIgA were significantly increased in DSS-treated mice (*p <* 0.05), and the levels of MPO, TNF-α, IL-1β, and IL-6 were effectively decreased (*p <* 0.05). Additionally, the Akk EVs group exhibited significantly higher IgA and sIgA than the DSS group (*p <* 0.05). Meanwhile, TNF-α, IL-1β, IL-6, and MPO levels in the Akk EVs group were significantly lower than that of the DSS group (*p <* 0.05). Akk and Akk EVs showed similar effects. These findings indicated that the regulation of intestinal inflammation by Akk EVs correlated with the activation of immunoglobulins and suppression of pro-inflammatory cytokines.

### 3.7. Effects of Akk EVs on Intestinal Permeability and Barrier Function

**Figure 7:**
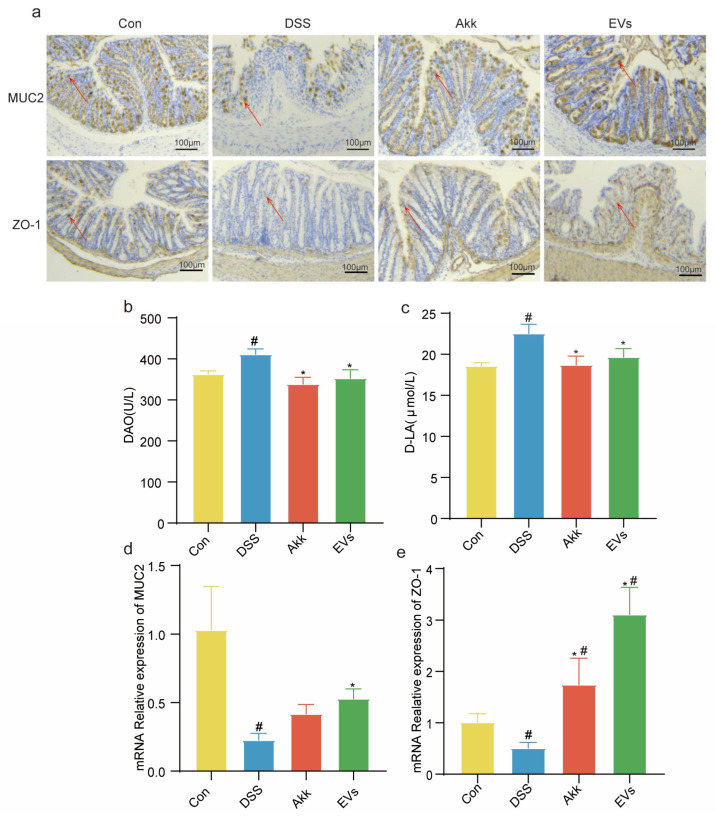
Effects of Akk EVs on intestinal permeability and barrier function. (**a**) Images of immunohistochemistry of MUC2 and ZO-1. Scale bar represents 100 μm. (**b**) Concentration of DAO. (**c**) Concentration of D-LA. (**d**) mRNA expression of MUC2. (**e**) mRNA expression of ZO-1. All data were presented as mean ± SD (*n* = 5). Red arrows indicated protein positive expression. Control group (Con), DSS group (DSS), Akk group (Akk), and Akk EVs group (EVs). * *p* < 0.05, compared with the DSS group; # *p* < 0.05, compared with the control group.

To examine the effects of Akk EVs on intestinal permeability and barrier function, the serum concentrations of DAO and D-LA and the alterations in tight junctions (MUC2, ZO-1) were measured. As shown in Figure 7b,c, in the control group, the concentrations of DAO and DLA were measured to be 363.30 ± 7.87 U/L and 18.59 ± 0.38 µmol/L, respectively. In the DSS group, these concentrations were found to be 411.30 ± 13.27 U/L and 22.54 ± 1.11 µmol/L, respectively. In the Akk group, the concentrations of DAO and DLA were determined to be 339.0 ± 16.22 U/L and 18.71 ± 1.06 µmol/L, respectively. Similarly, in the EVs group, the concentrations of DAO and DLA were recorded as 353.0 ± 20.71 U/L and 19.69 ± 1.00 µmol/L, respectively. In comparison to the control group, DSS significantly increased the concentrations of DAO and D-LA. And Akk and Akk EVs treatments exhibited markedly lower DAO and D-LA concentrations (*p <* 0.05). As shown in Figure 7a,d,e, the localization and mRNA relative expression of tight junctions (MUC2, ZO-1) in intestinal mucosa were investigated by immunohistochemistry and Quantitative Real-Time PCR. The positive and mRNA expression of tight junctions decreased in the DSS group compared to the control group. In the control group, the mRNA expressions of MUC2 and ZO-1 were measured to be 1.03 ± 0.32 and 1.01 ± 0.17, respectively. In the DSS group, these mRNA expressions were found to be 0.23 ± 0.05 and 0.51 ± 0.11, respectively. However, the tight junctions were increased to varying degrees after treatment with Akk and Akk EVs. Compared to the DSS group, the mRNA expression of MUC2 in the Akk group and Akk EVs group showed an increase to 0.42 ± 0.07 and 0.53 ± 0.07, respectively. Similarly, the mRNA expression of ZO-1 in the Akk group and Akk EVs group exhibited an increase to 1.75 ± 0.51 and 3.11 ± 0.52, respectively. These results revealed that Akk EVs could protect intestinal permeability and integrity to prevent damage to the intestinal barrier.

### 3.8. Effects of Akk EVs on Gut Microbiota

**Figure 8:**
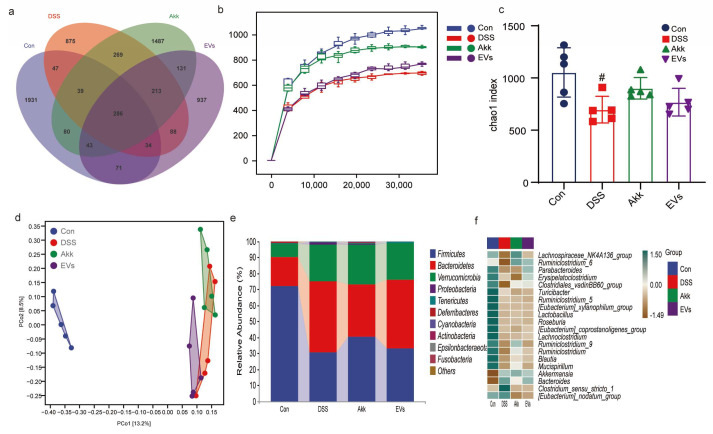
Effects of Akk EVs supplements on the gut microbiome structure. (**a**) Venn diagrams of OTUs. (**b**) Rarefaction curves. (**c**) Chao1 index. (**d**) PCoA analysis. (**e**) phylum level. (**f**) genus level. All data were presented as mean ± SD (*n* = 5). Control group (Con), DSS group (DSS), Akk group (Akk), and Akk EVs group (EVs). # *p* < 0.05, compared with the control group.

As shown in Figure 8a, 875 OTUs in the DSS treatment group were much lower than the control, Akk, and Akk EVs groups. There were 1931, 1487, and 937 OTUs in the control, Akk, and Akk EVs groups, respectively. In addition, the rarefaction curve reflected the total number of species and the relative percentage of each species in a given sequencing depth of a sample, thereby measuring the diversity of each sample to a certain extent. Figure 8b shows that the curve becomes flat with a high enough sequencing depth, indicating the sequencing data’s reliability. The DSS group had the lowest level, suggesting dysbacteriosis and a lower number of species than other groups. However, the Akk EVs group revealed a slight increase in trend. 

The α-diversity in Figure 8c showed a specific area’s microbial diversity and richness. The Chao1 index results showed that the α-diversity of the DSS group was significantly reduced compared with the control group (*p* < 0.05). The Akk and Akk EVs groups increased compared with the DSS groups, but no significant differences were observed.

To explore differences in gut microbiota composition among different groups, the β-diversity was measured, including principal coordinates analysis (PCoA) in Figure 8d. The results showed that the control group and the DSS group were far away from each other and that there was an apparent separation of gut microbiota composition between Akk EVs and the DSS group (Anosim, *p* < 0.05). Nevertheless, the Akk group was slightly separate from the DSS group.

As shown in Figure 8e, the alterations in the gut microbiota at the phylum level were analyzed in all groups. The four groups had a similar composition of gut microbiota and a distinct abundance of microorganisms. The dominant phylum were *Firmicutes* and *Bacteroides*, accounting for approximately 70% of the total. After DSS treatment, the relative amount of *Bacteroidetes* and *Proteobacteria* was increased, and the relative amount of *Firmicutes* was decreased compared with the control group. However, there was a reverse after treating Akk and Akk EVs. The *Firmicutes*/*Bacteroidetes* (F/B) ratio was 3.97, 0.69, 1.23, and 0.76 in the control, DSS, Akk, and Akk EVs groups. In contrast to the control group, the F/B ratio decreased as a result of DSS. However, the F/B ratio decreased less when Akk and Akk EVs were treated. At the genus level, DSS led to a decreased abundance of *Lachnospiraceae_NK4A136*, *Ruminiclostridium_6*, and *Parabacteroides* (Figure 8f), while Akk EVs treatment reversed this phenomenon.

**Figure 9:**
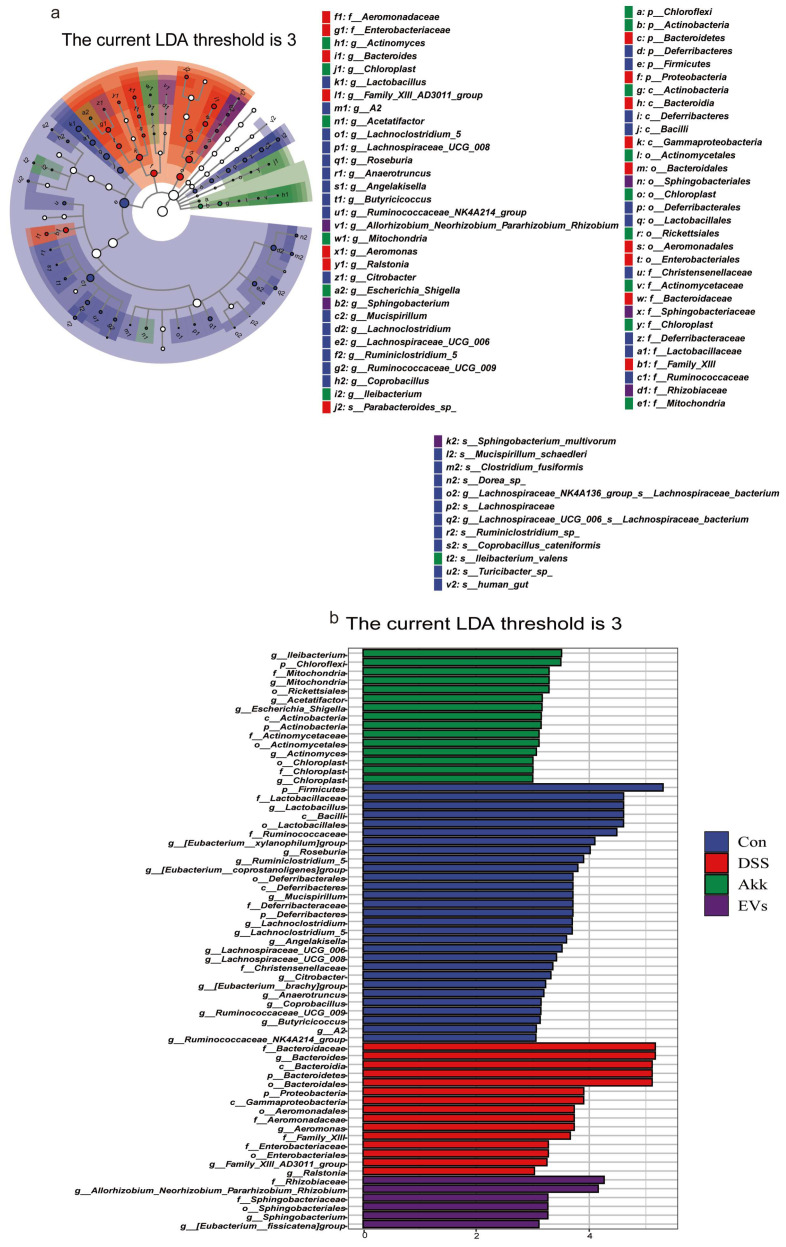
The effect of Akk EVs on gut dominant microorganisms. (**a**) The taxonomic tree of differentially abundant taxa based on genus was depicted by the cladogram. (**b**) Histogram of the distribution of dominant gut microorganisms at the genus level. Control group (Con), DSS group (DSS), Akk group (Akk), and Akk EVs group (EVs).

The effects of Akk EVs administration on the gut microbiome were estimated using linear discriminant analysis (LDA) and linear discriminant analysis effect size (LDfSe) comparison analysis in order to examine the variations in gut microbiota across the four groups (Figure 9a,b). The results illustrated that the dominant was *Bacteroidales*, *Aeromonadales*, and *Enterobacteriales* in the DSS group, yet *Sphingobacteriales* were dominant in the EVs group.

Through the analysis of gut microbiota, these results illustrated that the richness and diversity of the gut microbiota of mice were improved after the administration of Akk EVs, which suggested that Akk EVs had the potential to enhance the presence of probiotic bacteria and decrease the presence of pathogenic bacteria.

## 4. Discussion

IBD is characterized by chronic inflammation and relapsing-remitting disorders of the large bowel [27]. The global incidence and prevalence of IBD are increasing annually, and approximately 7 million people worldwide are suffering from it [28]. IBD seriously affects patients’ quality of life, and the incidence tends to be younger [29]. Numerous researches conducted in the last few years have suggested that the intestinal barrier may be a viable treatment target for IBD [30]. 

Akk, as a next-generation probiotic, was reported to effectively regulate human health, including metabolic regulation, immune modulation, and gut health protection [31]. Multiple studies have shown that Akk alleviates colitis by regulating the intestinal barrier. It was reported that Akk activated NLRP3 [32], upregulated RORγt^+^ Treg cells [33], and improved the microbial community [34] to alleviate colitis. It was consistent with previous results that treatment with Akk prevented DSS-induced colitis in our study. 

BEVs are spherical lipid bilayer nanostructures between 20 and 400 nm in diameter that contain proteins and small molecules [35]. BEVs are essential mediators of bacteria-host communication and bacteria-bacteria interaction, playing a significant role in various physiological activities and disease processes [36]. For example, Biomineralized bacterial extracellular vesicles [37] could exert a positive impact on the host. In addition, studies have shown that Akk outer membrane vesicles could enter intestinal epithelial cells to stimulate tight junction and mucus expression to maintain the integrity of the intestinal barrier [14]. Consistent with previous studies, we revealed that Akk EVs could prevent DSS-induced mouse colitis by modulating the intestinal barrier, which included the physical barrier, chemical barrier, immune barrier, and biological barrier.

Currently, there is no unified standard for the extraction method for microbe-derived extracellular vesicles. In this study, we established a method combining ultrafiltration and ultracentrifugation to extract Akk EVs. The diameter of the extracted Akk EVs was between 30 and 350 nm, and the morphology of EVs was a sphere with a double-layer membrane structure. These morphological characters of Akk EVs were consistent with the previous reports [16].

Previous research [38] found that EVs released by Gram-negative bacteria could be taken in by host cells and change the immune system of the host by delivering bacterial effectors and immunomodulatory small RNAs (sRNAs) from their parent bacteria or by causing damage to mitochondria. In this study, we found that Akk EVs could effectively be internalized by RAW264.7 cells, which was in line with the previous results [39]. In addition, we observed that the levels of NO, IL-1β, and TNF-α secreted by LPS-treated RAW264.7 cells were significantly higher than those of the control group. After Akk EVs treatment, the levels of pro-inflammatory cytokines were reduced. These findings indicated that Akk EVs could be internalized by RAW264.7 cells, thereby regulating the immune response and improving the abnormal secretion of inflammatory cytokines caused by LPS stimulation.

In the research, a mouse model of colitis using 3.5% DSS was established. The clinical symptoms, including bodyweight loss, DAI score increase, colon shortening, and colonic tissue damage, conformed to the research from Samuel C. Forster [40]. However, supplementation with Akk EVs reversed these changes. On day 28, the body weight loss and the DAI score of the Akk EVs group were markedly lower than those of the DSS group. In addition, the DSS group had a substantially shorter colon length than the control group, whereas the Akk EVs group had a longer colon length than the DSS group. The epithelial structure of the colon surface was damaged, including inflammatory cell infiltration, goblet cell depletion, and mucus layer reduction in the DSS group. After Akk EVs intervention, the colonic inflammatory cell infiltration abated, the number of goblet cells was raised, and the mucus layer was restored. These findings implied that supplementing Akk EVs could effectively improve the symptoms of colitis. Similarly, previous studies also showed that Akk EVs had versatility in regulating intestinal homeostasis [16]. Therefore, Akk EVs could be expected to be potential prebiotics or postbiotics for the prevention of IBD. 

Intestinal antigens like food, harmful bacteria, and enteric pathogens are exposed to innate and adaptive immune cells in the intestine during the progression of colitis due to the loss of the intestinal mucus layer and the reduction in tight junctions, which increases intestinal permeability and disrupts the intestinal barrier [41]. This ultimately causes intestinal inflammation. BEVs play essential roles in immune regulation and the protection of gut barrier integrity [42].

It was observed that a layer of protective mucus was secreted by goblet cells on the surface of the intestinal tract to prevent environmental antigens from directly invading the intestinal wall tissue [43]. MUC2 is the principal constituent of this mucus layer [44]. Goblet cells could produce a specific sialyltransferase (ST6GalNac, ST6) that determined the sialylation of MUC2. MUC2 sialylation could prevent intestinal mucosa from mucinase degradation by bacteria and protect the intestinal barrier [45]. Therefore, we speculated that Akk EVs could protect the intestinal tract by raising the amount of goblet cells, promoting the production of specific sialyltransferase and increasing the sialylation of MUC2. It was consistent with the increase in the number of acidic mucins observed by AB-PAS staining.

ZO-1 plays a crucial role in forming a barrier at the apical site of adjacent epithelial cell membranes, effectively preventing the paracellular transport of molecules between cells. Additionally, adherens junctions, located basolaterally but subjacent to tight junctions, facilitate interactions among adjacent epithelial cells. It helps sustain the integrity of the barrier and aids in its closure [46]. The decrease in ZO-1 expression caused the destruction of the intestinal barrier. The pathogenic bacteria would pass through the epithelium to reach the lamina propria, activate macrophages and NK cells, and release a large number of pro-inflammatory factors [47]. Consequently, intestinal barrier dysfunction would reduce intestinal permeability, and some macromolecular substances such as D-LA and DAO would exudate into the blood [48]. Therefore, it was assumed that Akk EVs could protect the intestinal barrier and reduce inflammation because they could enhance the expression of ZO-1.

It is widely accepted that gut microbes play a vital part in maintaining intestinal balance [49] and are closely related to human health or disease pathogenesis [50]. It had been reported that IBD was frequently linked with a reduction of bacterial diversity, an alteration of the gut microbiota, a drop in *Firmicutes* [51], and a rise in *Proteobacteria* [52]. The seriousness of IBD is strongly linked to the increase in *Proteobacteria*, which includes many harmful bacteria, such as *Enterobacteriaceae* [53]. A previous study showed that *Enterobacteriaceae* was enriched in patients with colitis and *Sphingobacteriales* in healthy people [54]. Similar to the results of a previous study, we revealed that *Enterobacteriaceae* was effectively enriched in colitis mice and *Sphingobacteriales* was enriched in the Akk EVs group. It indicated that Akk EVs promoted the recovery of colitis mice. *Firmicutes* were the major bacterial phylum colonizing the healthy human gut, including *Lachnospiraceae_NK4A136*, and *Ruminiclostridium_6*, which may cooperate with the mucosa of the intestine to support intestinal homeostasis [55]. We found that Akk EVs mainly increased the quantities of *Lachnospiraceae_NK4A136* and *Ruminiclostridium_6*. Related studies have found that *Lachnospiraceae_NK4A136* [56] and *Ruminiclostridium_6* [57] play an essential part in regulating intestinal barrier function, improving intestinal microbiota balance, and inhibiting colitis. It indicated that Akk EVs could increase the number of beneficial bacteria to regulate the intestinal barrier and relieve colitis. Furthermore, Wang et al. [14] discovered that Akk outer membrane vesicles could stimulate the proliferation of beneficial bacteria through selective fusion. Cao et al. [58] found that BEVs could inhibit specific pathogens by membrane fusion. Teng et al. [59] reported that plant-derived extracellular vesicles could be selectively absorbed by *Lactobacillaceae*, leading to the regulation of mRNA and protein expression and subsequent modulation of the intestinal flora structure. Based on these findings, Akk EVs may be absorbed by specific intestinal bacteria and subsequently regulate the structure of the intestinal flora by modulating mRNA and protein expression. However, the mechanism by which Akk EVs regulate mRNA and proteins in specific intestinal bacteria remains to be explored.

**Figure 10:**
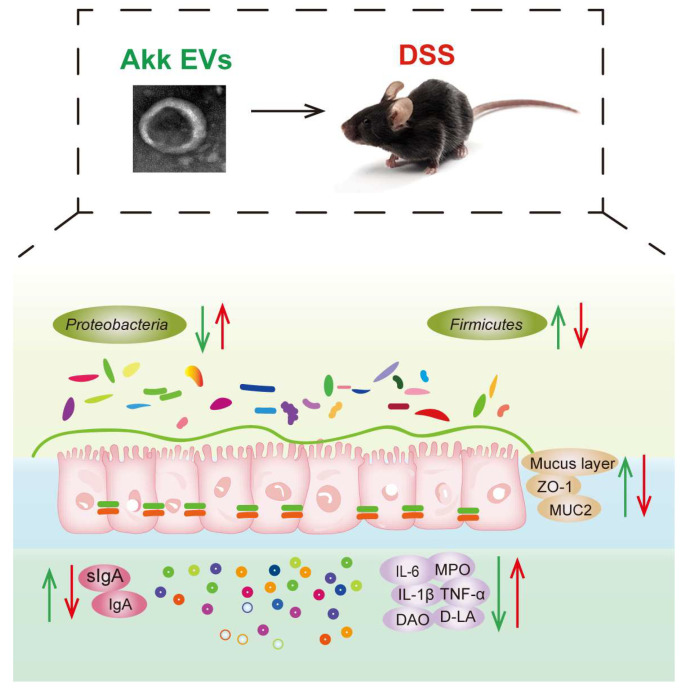
Schematic illustration of Akk EVs regulating intestinal barrier.

Although a similar probiotic effect was observed between Akk and Akk EVs, Akk EVs possess a stronger advantage and greater value than Akk. EVs are nano-sized natural vesicles with the ability to traverse the blood-brain barrier, providing a unique advantage for disease treatment [60]. In addition, EVs exhibit great biocompatibility and can specifically target cells and tissues, making them an excellent carrier for drug delivery [61]. It is critical to recognize that BEVs are a promising replacement for bacteria. However, it is important to remember that the research on BEVs is still in its infancy. Collectively, our investigations implied that Akk EVs could effectively prevent intestinal inflammation in mice with colitis by modulating the immune response, protecting intestinal barriers, and modulating the intestinal microbiota. As shown in Figure 10, Akk EVs have been discovered to preserve the intestinal barrier and inhibit the exudation of DAO and DLA by increasing mucin content and promoting ZO-1 expression. Additionally, Akk EVs enhance the secretion of IgA and sIgA while inhibiting the secretion of TNF-α, IL-6, IL-1β, and MPO. These effects are attained through the regulation of intestinal immune response and the structure of the gut microbiome.

## 5. Conclusions

The probiotic effect of Akk EVs was investigated in vivo and in vitro. We utilized LPS to induce an inflammatory model using RAW264.7 cells in vitro and discovered that Akk EVs could be absorbed by RAW264.7 cells, exerting an anti-inflammatory effect. The preventive and protective effects of Akk EVs on colitis were evaluated in vivo. It was revealed that oral administration of Akk EVs for a duration of 3 weeks significantly inhibited weight loss and colon shortening in colitis mice. He staining and AB-PAS staining demonstrated that Akk EVs reduced colonic pathological changes and mucus depletion. Additionally, supplementation with Akk EVs decreased the pro-inflammatory factors, improved intestinal permeability, and increased the expression of tight junctions MUC2 and ZO-1. Akk EVs regulated the balance of intestinal microbiota with an elevated quantity of *Firmicutes* and a reduced quantity of *Proteobacteria*. These comprehensive findings highlighted that Akk EVs could prevent colitis by modulating the intestinal barrier, immune response, and intestinal flora, thereby presenting as promising prebiotics or postbiotics.

## Data Availability

The initiative is still collecting data, but all research data can be obtained by contacting the authors.

## Supplementary Materials

The following supporting information can be downloaded at: https://www.mdpi.com/article/10.3390/nu15224722/s1, Table S1: Criteria for disease activity index (DAI) scoring. Table S2: Scoring standard of colon histological injury. Table S3: Primer sequence for RT-PCR analysis.
