# Supplementary material for: Effect of Extracelluar Vesicles Derived from Akkermansia muciniphila on Intestinal Barrier in Colitis Mice
Source: Nutrients. 2023 Nov 8;15(22):4722. doi: 10.3390/nu15224722 (PMC10674789; doi:10.3390/nu15224722)
Supplement: Supplementary file 1 [file nutrients-15-04722-s001.zip › nutrients-2680522-supplementary.pdf]

Supporting information

Table S1. Criteria for disease activity index (DAI) scoring.

Score	Weight	Stool Consistency	Stool Bleeding
0	None	Normal	Negative hemocult
1	1-5%	Soft but still formed	Negative hemocult
2	6-10%	Soft	Positive hemocult
3	11-15%	Very soft; wet	Blood traces in stool visible
4	>15%	Watery diarrhea	Gross retal bleeding

Table S2. Scoring standard of colon histological injury.

Score	Inflammation Severity	Inflammation Extent	Crypt Damage	Percent Involvement
0	None	None	None	None
1	Mild	Mucosa layer	1/3 of base crypts destroyed	1-25
2	Moderate	Submucosa	2/3 of base crypts destroyed	26-50
3	severe	Muscle layer	Only intact surface epithelium	51-75
4	-	-	All crypts and epithelium were destroyed	76-100

Table S3. Primer sequence for RT-PCR analysis.

Gene	Forward Pimer Sequence (3\`-5\`)	Reverse Primer Sequence (5\`-3\`)
GAPDH	GTGGAGTCATACTGGAACATGTAG	AATGGTGAAGGTCGGTGTG
MUC2	TGCTGACGAGTGGTTGGTGAATG	TGATGAGGTGGCAGACAGGAGAC
ZO-1	CTGATGGTGCTCTGCCTAAT	TAGTCGCAAACCCACACTATC
